# Refining transcriptional programs in kidney development by integration of deep RNA-sequencing and array-based spatial profiling

**DOI:** 10.1186/1471-2164-12-441

**Published:** 2011-09-05

**Authors:** Rathi D Thiagarajan, Nicole Cloonan, Brooke B Gardiner, Tim R Mercer, Gabriel Kolle, Ehsan Nourbakhsh, Shivangi Wani, Dave Tang, Keerthana Krishnan, Kylie M Georgas, Bree A Rumballe, Han S Chiu, Jason A Steen, John S Mattick, Melissa H Little, Sean M Grimmond

**Affiliations:** 1 Institute for Molecular Bioscience, The University of Queensland, St. Lucia QLD 4072, Australia

**Keywords:** RNA-Seq, kidney development, microarray, *Six2, Wt1*, sense-antisense transcripts, alternative splicing, mesenchymal-epithelial transition, miR-214, microRNA

## Abstract

**Background:**

The developing mouse kidney is currently the best-characterized model of organogenesis at a transcriptional level. Detailed spatial maps have been generated for gene expression profiling combined with systematic *in situ *screening. These studies, however, fall short of capturing the transcriptional complexity arising from each locus due to the limited scope of microarray-based technology, which is largely based on "gene-centric" models.

**Results:**

To address this, the polyadenylated RNA and microRNA transcriptomes of the 15.5 dpc mouse kidney were profiled using strand-specific RNA-sequencing (RNA-Seq) to a depth sufficient to complement spatial maps from pre-existing microarray datasets. The transcriptional complexity of RNAs arising from mouse RefSeq loci was catalogued; including 3568 alternatively spliced transcripts and 532 uncharacterized alternate 3' UTRs. Antisense expressions for 60% of RefSeq genes was also detected including uncharacterized non-coding transcripts overlapping kidney progenitor markers, Six2 and Sall1, and were validated by section *in situ *hybridization. Analysis of genes known to be involved in kidney development, particularly during mesenchymal-to-epithelial transition, showed an enrichment of non-coding antisense transcripts extended along protein-coding RNAs.

**Conclusion:**

The resulting resource further refines the transcriptomic cartography of kidney organogenesis by integrating deep RNA sequencing data with locus-based information from previously published expression atlases. The added resolution of RNA-Seq has provided the basis for a transition from classical gene-centric models of kidney development towards more accurate and detailed "transcript-centric" representations, which highlights the extent of transcriptional complexity of genes that direct complex development events.

## Background

The mammalian kidney is a remarkably complex organ at the cellular and functional level, being essential not merely for excretory functions but also for a variety of hormonal and homeostatic regulatory functions. A key structure is the nephron, which represents the functional excretory units of the kidney. During kidney development, the nephron arises via a reciprocal interaction between a mesenchymal progenitor population and an adjacent epithelial ureteric tip, where the latter induces the former to undergo a mesenchymal-to-epithelial transition (MET), signaling the start of nephrogenesis (reviewed in [1,2]). Although well studied, the complete transcriptional regulatory networks are just beginning to be elucidated.

Transcriptional profiling of the developing kidney using microarrays coupled with RNA *in situ *hybridizations (ISH) have provided a detailed view of gene expression networks driving developmental processes [3-6]. Despite these advances, microarrays cannot capture the entire transcriptional output from mammalian genes (reviewed in [7,8]) as they require *a priori *assumptions about the portion of the genome that is expressed, limiting the ability to use this technology for uncharacterized gene or transcript discovery [8]. This also applies to mRNA variants. On average, 6-7 different mRNA variants can arise from a single active locus [9], and this complexity includes alternate promoters, alternate 3' untranslated regions (UTRs), alternative exons, and alternative splice sites. The vast majority of this complexity is invisible to microarray probes, which are typically short (25-70 nt) and located in the 3' UTR of transcripts [10]. Such limitations mean that kidney developmental programs have only been explored at "gene-centric" resolution. Given the consequences of transcriptional complexity (alternate domain content, differential transcription factor binding sites and microRNA binding sites from alternative promoter and 3'UTR usage, respectively), understanding the complete repertoire of transcripts is crucial for accurate modelling of kidney organogenesis.

Massive-scale sequencing of transcriptomes (RNA-Seq) overcomes most of the limitations imposed by microarrays, and additionally offers high dynamic range, increased accuracy, and increased specificity [11-13], although not yet capable of single cell resolution. Application of this technology has enabled the identification of uncharacterized transcripts, genes, and non-coding RNAs (ncRNAs) [11,12,14,15], and in all studies, the level of complexity has been far higher than previously predicted. Although these features make it highly desirable, RNA-Seq is not practical for all experiments, due primarily to laborious protocols and the need for large quantities of starting material. The recent application of single-cell RNA-Seq has allowed profiling of samples with limited quantities of sample such as embryonic development, but this technique did not discriminate strand-specific transcripts and did not detect 5' ends of transcripts longer than 3 kb which would hinder analysis of alternative promoter usage [16,17]. For the analysis of complex processes such as organogenesis where individual cellular components are difficult to separate, RNA-Seq to this level of resolution is not practical whereas gene expression profiling on whole organs may fail to detect subcompartment specific transcripts. The integration of both types of analyses, however, may overcome the limitations of each, without the need of completely replacing current wealth of high-quality microarray datasets.

In this study, we describe a high quality, stranded, polyadenylated RNA-Seq and microRNA (miRNA)-Seq profiling resource of the whole embryonic mouse kidney for the purpose of integrating with previously defined spatial resolution kidney microarray. In comparison to the microarray kidney atlas [5], we show that high coverage whole organ RNA-Seq is sensitive enough to both detect compartment-specific transcripts, and quantify transcript abundance relative to the whole organ. We have used this technique to assess the transcriptional complexity within the developing kidney subcompartments, identifying mRNA variants of many key kidney developmental genes. We also detect wide-spread sense-antisense transcription among important MET regulators, which we validated by SISH. Together, the datasets generated in this study advance gene-centric models of kidney development pathways towards more complete transcript-centric models, capturing the transcriptional landscape of gene expression.

## Results

### Deep sequencing of the 15.5 dpc mouse kidney

The 15.5 dpc embryonic mouse kidney contains subcompartments representing all progression of states during renal development [5]. The total ribosomal-RNA depleted transcriptome (including miRNAs) of the 15.5 dpc mouse kidney was surveyed using massive-scale stranded sequencing on the SOLiD platform. Approximately 136 million high-quality, single mapping reads were mapped to the reference mouse genome (mm9) for the RNA-Seq library, and 788,931 uniquely mapping tags to known pre-miRNA hairpins (miRBase version 15 [18]; (Table 1). Datasets are accessible from NCBI Short Reads Archive (SRA026710)).

**Table 1:** RNA-MATE and Galaxy tag mapping distribution

Total tags	329,923,262
Total tags mapping to genome *(mm9)*	136,122,785 *(41.3%)*
Total unique tags	107,339,260 *(32.5%)*
Number of RefSeq genes (> 1RPKM)	12,083
Number of transcripts (> 1RPKM)	15,527
Unique tags matching RefSeq NM exons	66,591,988 *(62%)*
Unique tags matching consensus gene exons models (RefSeq, Aceview, Ensembl, UCSC genes)	82,841,356 *(77.1%)*
Total unique junction tags	7,769,426
Total unique miRNA tags	788,931

### Quantifying embryonic kidney locus activity

**Figure 1:**
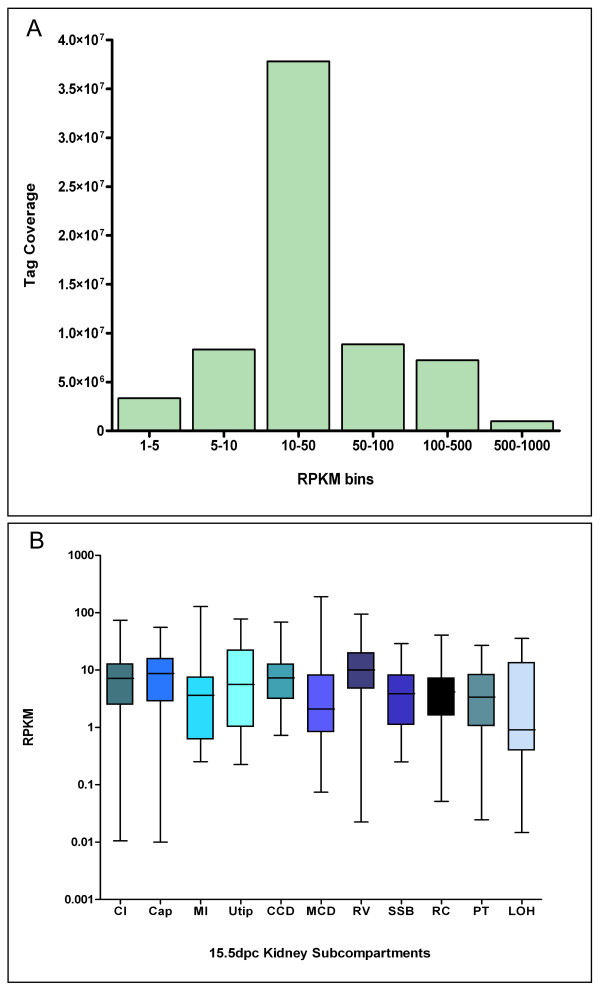
**Embryonic kidney RNA-Seq coverage, depth and sensitivity**. **A: **Tag distribution across active genes with varying levels of expression in the 15.5 dpc mouse kidney. Genes are grouped into reads per kilobases per million (RPKM) (y-axis) bins according to expression abundance based on tag coverage (x-axis). Low abundance genes are considered to have RPKM values between, 1-10 RPKM, moderate expression at 10-100 RPKM, and highly expressed at above 100 RPKM. **B: **Box-plot representation of embryonic kidney subcompartments captured by whole-kidney RNA-Seq profiling. Transcripts with the most subcompartment-specific expression from each structured identified from the embryonic kidney subcompartment microarray atlas (Brunskill et al. [5]) were represented by RPKM values (log 10) as detected by RNA-Seq to gauge sensitivity of detecting specific embryonic kidney cell-types. Each box represents kidney subcompartment-specific transcripts with corresponding RPKM values; The boxes extend from the 25^th ^percentile (lower hinge) to the 75^th ^percentile (upper hinge) of RPKM values. The line across the box represents the median. The lengths of the lines above and below the box are defined by the maximum and minimum RPKM values (respectively). Subcompartments: CI: cortical interstitium; Cap: cap mesenchyme; MI: medullary interstitium; Utip: ureteric tip; CCD: cortical collecting duct; MCD: medullary collecting duct; RV: renal vesicle; SSB: s-shaped body; RC: renal corpuscle; PT: proximal tubule; LOH: loop of Henle.

Sequenced Reads Per Kilobase per Million (RPKM) values [12] for RefSeq exon models were calculated and compared to a high-resolution kidney subcompartment microarray gene expression atlas [5]. 12,083 active protein-coding loci (RefSeq "NM" ID's only) above 1 RPKM were identified (Additional file 1). This compares to ~5,300 microarray probesets representing 4,248 RefSeq protein coding loci previously identified based on robust gene expression levels using the kidney subcompartment atlas [5]. The majority of active loci were expressed at moderate to high levels (10-50 RPKM) (Figure 1A) with many key kidney developmental genes detected within this range. For example, *Six2*, a marker of the nephron progenitor population [19,20] was detected at 25 RPKM, and *Wnt4*, a marker of renal vesicle, at 16 RPKM. Low-level expressing transcripts such as *Shh *can also be detected in our experiment, at 1 RPKM, which approximates to roughly 1-2 transcript per cell [12,21]. The RPKM standardization based on RNA-Seq tag count offers a sensitive and precise measure of transcript abundance relative to the whole organ.

### Detecting rare, tissue-specific transcripts

A major concern of whole organ profiling is the inability to detect rare, cell-type specific transcripts due to the heterogeneity of tissue composition [22]. In the previously described microarray kidney atlas, this was addressed by profiling individual kidney subcompartments [5]. Subcompartment specific transcripts from that kidney microarray atlas were used to determine the sensitivity of tissue-specific transcript detection in whole organ RNA-Seq. As many as 99.7% of all transcripts attributed to major kidney subcompartments were detected, where the remaining discordant probe-sets were prone to cross-hybridizations as noted by probe-set ID suffixes (_s_at, _x_at, and a_at_ [23]) or generally had low raw signal (below 100 Raw Fluorescent Units) and therefore may be affected by background signal.

In addition, subcompartment-specific transcripts provided the framework to estimate the overall distribution of expression within kidney subcompartments. As shown in Figure 1B, all major kidney subcompartments were represented, where the mean expression abundance for each compartment was between 1-10 RPKM. Rare (0.5 RPKM), subcompartment-specific transcripts detected by the kidney microarray atlas were also identified by RNA-Seq. This confirms that with sufficient sequencing depth, whole organ RNA-Seq can be used to detect gene expression that are representative of specific kidney cellular populations.

### Integration of RNA-Seq with spatially-resolved Affymetrix microarrays

**Figure 2:**
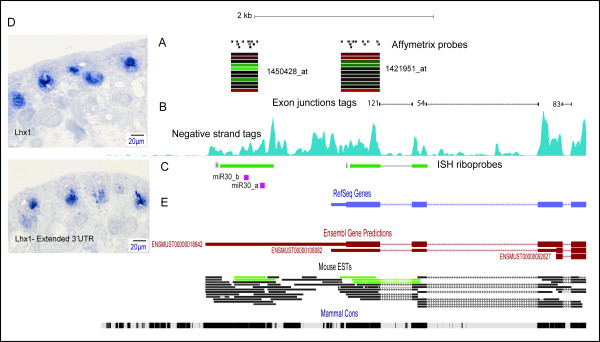
**Visualization of RNA-Seq and kidney subcompartments microarray data on UCSC Genome Browser of *Lhx1 *long 3'UTR**. Representation of the 3' end of the mouse *Lhx1 *gene (chr11:84330068-84335347) (mm9) is shown within the genome browser along with default and custom tracks. **A: **Affymetrix mouse 430.2 microarray platform probesets *1421951_at *localized to the canonical 3' untranslated region (UTR) and *1450428_at *~500 bp downstream; and corresponding probeset expression heatmap across kidney subcompartments (microarray data from [5]) microarray compartments from top to bottom of heatmap: ureteric tip; s-shaped body; proximal tubule; cortical, and medullary interstitium; medullary, and cortical collecting duct; renal corpuscle; cap mesenchyme; loop of Henle; renal vesicle. **B: **RNA-Seq exon junction tags are represented as UCSC Genome Browser BED data tracks (top) spanning exons, and 'wiggle' plots showing coverage of negative strand tags corresponding to *Lhx1 *expression (bottom). **C: **Riboprobes used for *in situ *hybridization (ISH): *i) *overlapping the canonical region as represented by Affymetrix probeset *1421951_at *and *ii) *overlapping extended 3' signal captured by RNA-Seq and probeset *1450428_at*, which also contains a microRNA binding site for miR-30 [28]. **D: **Histological 15.5 dpc mouse kidney section ISH (SISH) of canonical 3'UTR *(i) *and extended 3'UTR *(ii) *both detected in distal compartments of the renal vesicle. **E: **Pre-built UCSC genome browser data tracks of: (top-bottom) mouse RefSeq genes, Ensembl gene model predictions, mouse expressed sequence tags (ESTs). Green tags represent EST tags derived from kidney cDNA libraries, and evolutionarily conserved regions (black).

After demonstrating that the RNA-Seq data was highly sensitive, we then wanted to integrate it with the spatial-resolution embryonic kidney microarray atlas and interrogate the transcriptional complexity driving mouse kidney organogenesis. Affymetrix Mouse 430.2 probe sets were aligned against the mouse genome (mm9) to define the boundaries of captured expression. The probe set genomic coordinates were then used to overlay subcompartment specific expression as a heatmap-based UCSC data track (Figure 2A, B). This revealed presence of probe sets that can be used to capture expression beyond annotated gene boundaries, which provides excellent spatial resolution for events such as extended 3'UTR expression (Figure 2), while non-coding RNA transcripts can also be captured by multiple or previously unassigned probe sets (Additional file 2). Concurrent use of the UCSC Genome Browser heatmap tracks with RNA-Seq tracks therefore provides spatial identification for any transcriptional complexity which overlaps the microarray probes.

### Extensive use of extended 3'UTRs in embryonic kidney subcompartments

The 3' UTR contains *cis*-regulatory elements important for mRNA stability, degradation, subcellular localization and translation. Therefore, accurate characterization of 3'UTR boundaries can help identify key regulatory elements, such as microRNA (miRNA) binding sites. In order to identify expression beyond currently annotated 3'UTR boundaries, we used a sliding window to survey contiguous signal within a 20 kb radius from the annotated 3' end (excluding regions overlapping known RefSeq transcripts including ncRNAs). This approach identified over 1500 genes with 3'UTRs that extend well beyond the mouse RefSeq boundary. Extended UTR sequence genomic coordinates identified by RNA-Seq were obtained from mm9 using Galaxy [24] to determine if such events were novel or due to incomplete annotations. We found that 720 instances of these extended UTRs have been seen in RefSeq orthologs, often as part of the transcript of genomes with more complete annotations such as human RefSeq (hg18). Overall we find 532 transcripts with previously unannotated 3'UTR extensions, demonstrating the widespread nature of this transcriptional event in the embryonic kidney (Additional file 3).

We then asked whether extended 3'UTR expression was prevalent among genes critical for kidney development by focusing on genes involved during mesenchymal-epithelial transition (MET), which is a critical process for nephron development. Extended UTR expression was detected within the *Lhx1 *locus, a critical transcriptional regulator of nephron endowment [25,26] (Figure 2). A ~1.5 kb signal beyond the RefSeq annotated 3' end was detected and represented by probesets 1421951_at (canonical 3'UTR based on RefSeq models) and 1450428_at (extended UTR) with high concordant expression (Pearson correlation R = 0.932). Section ISH (SISH) also confirmed the concordant expression between the extended 3'UTR and the remaining portions of the transcript, localized to the nephron precursor structures (renal vesicle, s-shaped body and nephron tubules) (Figure 2C, D). SISH data and detailed annotations are available at [27]. Studies have described miRNA binding sites for miR-30 within the extended region of *Lhx1 *3'UTR, where miR-30 inhibits *Lhx1 *expression and therefore embryonic kidney differentiation [28] (Figure 2C). This region overlaps with the extended signal detected in our RNA-Seq data, highlighting the importance of accurate representation of gene boundaries.

### Alternate exon usage associated with key kidney development loci

Large scale identification of alternative splicing is an essential pre-requisite that will facilitate important downstream functional characterization on how genes are regulated in a tissue-specific manner and the roles of alternate isoforms during developmental states. Alternative splicing can alter mRNA through a variety of mechanisms, including the addition and removal of exons, thereby affecting protein functional domain composition [29]. To identify the presence of isoforms associated with alternate exon usage, reads were mapped to a predefined library of known exon junctions sequences, as described in [11,30]. Results from the mapping revealed 3568 loci (> 1 RPKM) where alternate exon-junctions were detected (Additional file 4).

To gauge our effectiveness in detecting transcriptional complexity arising from key loci, we reviewed the transcriptional output from key kidney development genes and detected previously known variants (Table 2). For example, *Ret *isoforms, *Ret51 *and *Ret9 *which have different temporal requirements during the developing kidney, were identified through tags spanning exon-exon junctions and expression tags, where differential expression was observed at the C-terminal tails as previously reported [31] (Additional file 5).

**Table 2:** Transcriptional complexity and discovery across regulators of kidney development

Gene	Known variants (mouse/human)	Variant Junction Location	Type	Supporting transcript models	Number of tags
*Pax2*	**NC**	**-**	**Alt. 5'/Promoter**	**-**	**Signal**
	Pax2a/b	chr19:44865283-44890407	Cassette Exon	ENSMUST00000111979	13
		chr19:44831917-44835374	Donor/Acceptor	Pax2.bSep07	13
	**NC**	**chr19:44909958-44910469**	**Donor/Acceptor**	**N/A**	**10**

*Wt1*	**NC**	**chr2:104973652-105003491**	**Skip exon 4 &5**	**ENSMUST00000111100**	**10**
	Wt1 -exon 5	chr2:104983310-105003491	Skip exon 5	ENSMUST00000111101	350
	+/- KTS	chr2:105010157-105012389	Donor/Acceptor	ENSMUST00000139585	83

*Sall1*	Isoform A (long) & B (short)	chr8:91557288-91566260	Alt. 5'/promoter	A: (hg19)NM_002968 B: (hg19)NM_001127892	9
	**NC**	**chr8:91566334-91567384**	**Overlapping Exon**	**Sall1.dSep07**	**5**

*Eya1*	**NC**	**chr1:14294546-14294663**	**Alt. Exon**	**Eya1.fSep07**	**3**
	Isoform 1-4	chr1:14273270-14294515	Cassette Exon	ENSMUST00000080664	10
		chr1:14260914-14264155	Donor/Acceptor	ENSMUST00000027066	32
		chr1:14264215-14264624	Donor/Acceptor	Eya1.hSep097	4
		chr1:14264279-14264624	Donor/Acceptor	Eya1.aSep07	35

*Gdnf*	Isoform 1-2	chr15:7760047-7787580	Alt. 5'/promoter	Gdnf.aSep07	Signal
		chr15:7765678-7784357	Donor/Acceptor	Gdnf.bSep07	5

*Ret*	Ret51 (long)	chr6:118104019-118105315	Retained intron	NM_009050	4
	Ret9 (short)	-	Overlapping Exon	NM_001080780	Signal

*Wnt11*	Isoform A & B	chr7:105983621-106002321	Alt. promoter	Wnt11.cSep07	Signal
		chr7:105987691-105994975	Donor/acceptor	Wnt11.cSep07	5

*Bmp7*	**NC**	**chr2:172693513-172766073**	**Alt. exon**	**Bmp7.aSep07**	**Signal**

*Pax8*	**NC**	**chr2:24298651-24300095**	**Donor/Acceptor**	**ENSMUST00000129538**	**3**
	Isoform C	chr2:24291401-24291977	Donor/Acceptor	ENSMUST00000102940	8

*Six2*	**NC**	**chr17:86084844-86086736**	**Donor**	**N/A**	**6**

*Fgf8*	Isoform 2 & 3	chr19:45816160-45816410	Cassette Exon	NM_001166361; NM_001166362	4

*Wnt4*	**NC**	**chr4:136845255-136851407**	**Acceptor**	**Wnt4.bSep07**	**4**

(NC- not characterized)

**Figure 3:**
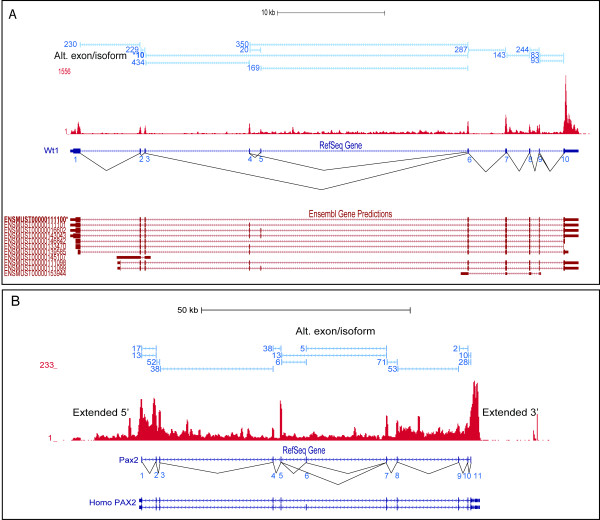
**Transcriptional complexity of kidney development regulatory genes**. **A: **Evidence of known and novel exon splicing in *Wt1 *positive strand. Exon junctions tags (> 3 tags) representing differential exon usage. Novel splicing event involving exons 4 and 5 is marked with '*'. Canonical RefSeq and supporting Ensembl gene models of predicted isoforms are shown. **B: ***Pax2*-locus with spliced exon 6, represented by exon junction tags (> 3 tags), resembling *PAX2 *RefSeq human isoform, shown below the mouse RefSeq track. Expression beyond mouse RefSeq gene boundaries was also captured. Exons are numbered below mouse RefSeq models.

In addition, uncharacterized splicing events were also detected. In *Wt1*, two main splicing events have been previously identified and characterized: splicing of exon 5 and exon 9 +/-KTS domain [32]. Together with three known alternate transcriptional start sites, up to 24 *Wt1 *protein isoforms are predicted with the ratio of isoform abundance proposed to be critical for normal development [33]. The RNA-Seq dataset detected both previously described alternate splicing events together with a novel isoform lacking both exons 4 and 5 [Ensembl Transcript: ENSMUST00000111100, Ensembl protein: ENSMUSP00000106729] (Figure 3A), where expression has been confirmed by qRT-PCR (Additional file 6). Previously, isoforms lacking exon 4 have only been reported in kidneys of aquatic/semi-aquatic animals including eel, medaka, and turtle [34-36] with such isoforms proposed to represent an event no longer required for mammalian metanephric kidney development. Our data would question this conclusion. Alternate donor-acceptor splice sites (GT-AG) across exon junctions were also detected among key kidney development regulators such as *Six2 *and *Wnt4*)See (Table 2).

### Temporo-spatial loci with uncharacterized 5' exons and alternative promoter signal

**Figure 4:**
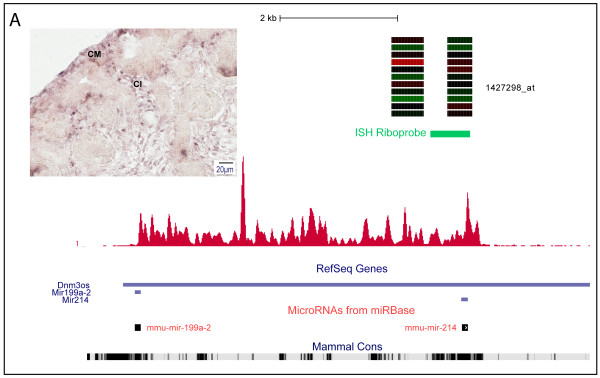
**Mesenchyme-specific expression of host gene *Dnm3os *for miR-214**. *Dnm3os *ncRNA host gene for microRNAs, miR-199 and miR-214. **A: **Affymetrix probeset *1427298_at *directly overlaps miR-214. Microarray compartments from top to bottom of heatmap: ureteric tip; s-shaped body; proximal tubule; cortical, and medullary interstitium; medullary, and cortical collecting duct; renal corpuscle; cap mesenchyme; loop of Henle; renal vesicle. *In situ *hybridization (ISH) riboprobe was designed to capture the exact expression detected by the Affymetrix probeset **B: **Section ISH images of *Dnm3os *show mesenchymal-specific expression including cap mesenchyme.

Alternative promoters, including those associated with alternate 5'exon usage, can be activated in a tissue-specific manner. For example, a Nephrin (*Nphs1*) isoform with exon 1a is detected in kidney and plays an important role in renal filtration [37] while the variant with exon 1b is only detected in brain [38]. Presence of alternative promoters associated with key temporo-spatial kidney development loci warrant further subsequent experimental validation to determine its potential role during gene expression regulation. To identify alternative promoters, the most 5' exon junction tags beyond the RefSeq gene models were screened for evidence of alternate or complex promoter usage. A minimum cutoff of 10 tags at each candidate junction was required which returned a total of 374 alternate exons associated with 187 genes (Additional file 7). Alternative 5' usage was detected among four key kidney development regulators (Table 2); including a shorter novel promoter for *Sall1*, an early inducer of kidney development, supported by RNA-Seq signal (See Figure 4B). Alternative 5' exon junctions in *Sall1 *were also detected, and this 5' complexity could be due to the multiple expression sites of this gene. *Sall1 *expression is detected during initial stages kidney development and subsequently expressed in nephron progenitors, but also in the and the subsequently formed early nephron epithelium [39]. Extended promoter signal ~12 kb beyond the RefSeq annotated start site was also detected for *Pax2 *(Figure 3B) which is expressed in both the ureteric epithelium and mesenchyme [40]. This promoter region encompasses a 4.1 kb minimal promoter that is only expressed in ureteric bud epithelia [41]. As the prediction of transcription factors (TFs) that regulate a cohort of genes requires the precise determination of the potential promoter region, using the standard promoter regions based on RefSeq gene models in these analyses may lack sensitivity. Incorporation of this RNA-Seq derived information into TF binding site predictions should uncover TF regulators of importance to the developing kidney and also aid in the design of promoter-reporter green fluorescent protein (GFP) constructs in transgenic mice to understand mechanisms regulating tissue- and cell- specific expression.

### Sequencing of embryonic kidney miRNAs

MiRNAs are short, non-coding species of RNA (~22nt) that function as translational repressors of target mRNAs during many biological processes including development, differentiation, cell proliferation and disease [42,43]. Within the kidney, tissue-specific knockout of Dicer, an enzyme required for miRNA biogenesis, has previously been reported to alter anatomical organization and to also play a role in renal diseases [44-46]. Identification of the complete miRNA repertoire in the embryonic kidney will serve as an important reference of developmentally regulated miRNAs for functional characterization. To catalogue active miRNAs within the developing mouse kidney, we have isolated and sequenced the small RNA fraction (SOLiD, Applied Biosystem) and mapped the reads against the entire miRBase (v15) database [18]. This provided the identification of over 170 microRNA families with high quantity of mapped tags (> 100 tags) (Additional file 8). MiR-30 was abundantly detected in our miRNA-Seq dataset, where it has been previously shown to be a critical regulator of kidney development [28]. The miR-200 family was also abundantly detected in the embryonic kidney which is likely due to its role in MET regulation [47,48]. Functional characterization of many more of kidney miRNAs identified by miRNA-Seq will be required to infer roles during organogenesis.

### Mesenchymal-specific expression of miR-214/*Dnm3os *in the developing kidney

One of the first steps to gain insights into the biological role of miRNAs is to determine tissue localization. SISH studies based on mature miRNA sequence hybridizations can be challenging due to the limited unique sequence content of these short molecules. To overcome this, several studies have described using miRNA precursor genes, known as primary transcripts (pri-miRNA), as a proxy to monitor expression of nested miRNAs [49,50]. Kidney miRNAs from the miRNA-Seq data were matched to corresponding intergenic noncoding pri-miRNAs (as annotated by Saini HK et al. [51]), that was also expressed in the mRNA-Seq data. We identified 22 highly expressed intergenic pri-miRNAs hosting kidney miRNAs including the Wilms tumor (renal neoplasm)-associated and imprinted transcript, *H19*, [52] a precursor for mir-675 [53] and the mir-17-92 cluster *Mirhg1 *pri-miRNA, with the latter being involved in embryonic lung proliferation and differentiation [54] (Additional file 9).

Next, we identified pri-miRNAs that were represented by Affymetrix 430.2 probeset from the kidney subcompartment atlas microarray data (Additional file 10). Of these probesets, three were co-incidentally positioned to overlap the embedded miRNAs within the primary transcript (let-7b:1440357_at; miR-425:1459927_at; miR-214: 1427298_at). Of these, miR-214 from the *Dnm3os *host gene provided the most reliable probe set expression profile. *Dnm3os *has been described to serve important roles during embryo development [55,56] although it has never been described within the context of the kidney. Micorarray probeset expression was detected in all interstitial mesenchyme subcompartments except the *Six2*^+ ^nephron progenitor population (Figure 4B). SISH validation of *Dnm3os*/miR-214 confirmed the interstitial mesenchyme specific expression profile but was also detected in the cap mesenchyme (Figure 4B and [GUDMAP:10816]). Further validation will be required to determine which cellular population of the cap mesenchyme miR-214 is restricted to and whether it is distinct from the *Six2 *population.

### Widespread expression of sense/anti-sense transcripts pairs in the embryonic kidney

The strand specific information of our RNA-Seq data enabled a genome-wide survey of sense-antisense transcription. Overlapping sense and antisense transcription has been described in a variety of biological roles, including RNA editing, genomic imprinting, translational regulation, RNA interference [57-60]. Current lists of validated sense-antisense pairs include many important developmental genes such as *Pax2 *and *Hoxa11 *[61]. Within the kidney, the noncoding antisense WT1 transcript (WT1-AS) shares the same expression domains as WT1 and therefore is consistent with its role as a positive regulator of WT1 protein levels [62]. Many splice-forms of WT1-AS have been characterized, where defects in the splicing machinery are implicated with acute myeloid leukaemia [63]. Survey of sense-antisense transcript pairs in the 15.5 dpc kidney identified 59.7% of expressed RefSeq transcripts with corresponding coding and non-coding antisense partners (Additional file 11) where only 2654 have been previously documented in the Natural Antisense Transcript Database (NATsDB) [64]. Antisense transcripts were detected for several kidney developmental genes, including *Wt1 *[62], *Sall1, Pax2 *[65]*Lhx1, Six2, Hnf1b, Emx2 *[66] and *Wnt7b*, where the majority overlapped in a head-to-head orientation. Examples of tail-to-tail (*Wnt9b*) and embedded overlaps (*Tcf21*) were also detected. Only a few of these kidney development antisense transcripts (e.g *Lhx1*) were represented on the Affymetrix platform.

**Figure 5:**
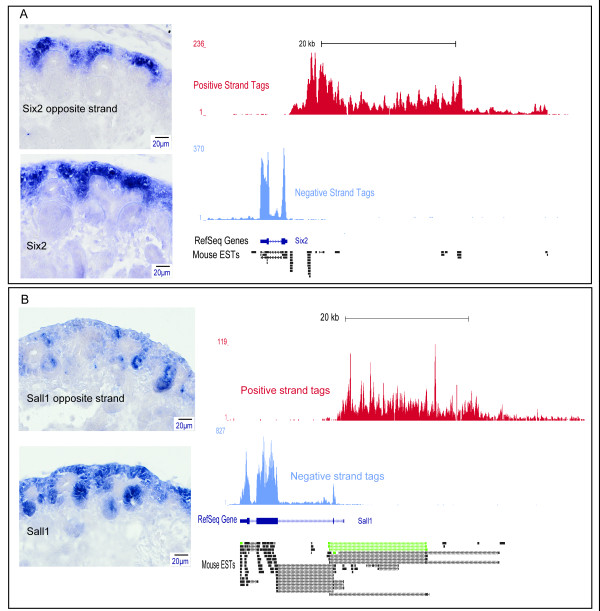
**Histological sections ISH (SISH) comparative analyses of sense and uncharacterized antisense transcripts expression**. SISH validations of: **A: ***Six2 *uncharacterized antisense (top) and sense transcript (bottom) in the cap mesenchyme; **B: ***Sall1 *antisense (top) and sense transcript (bottom), Supporting evidence of antisense expression from mouse EST tags. Green tags correspond to tags obtained from kidney-specific cDNA libraries.

To determine if antisense transcripts were spatially associated with the kidney development-associated sense transcript counterpart, high resolution SISH was performed on a small subset of these candidates. All three antisense transcripts for *Six2, Sall1*, and *Lhx1 *showed correlated subcompartment expression to sense counterpart although possibly at varying levels of intensity (Figure 5 and see also [GUDMAP:8504] for *Lhx1 *antisense (*1500016L03Rik*) validation). The previous association between head-to-head orientation and positive regulation of expression would agree with the higher intensity of expression of both sense and antisense *Sall1 *expression in the early nephrons as opposed to the lower levels of expression in the cap mesenchyme nephron progenitors (Figure 5B). Detailed annotations of SISH images are available at [27]. The identification of antisense transcription further validates the prevalence of natural antisense transcription in the genome [60], and is likely to contribute to the regulation of kidney developmental programs.

### Transcriptional complexity during mesenchymal-epithelial transition

**Figure 6:**
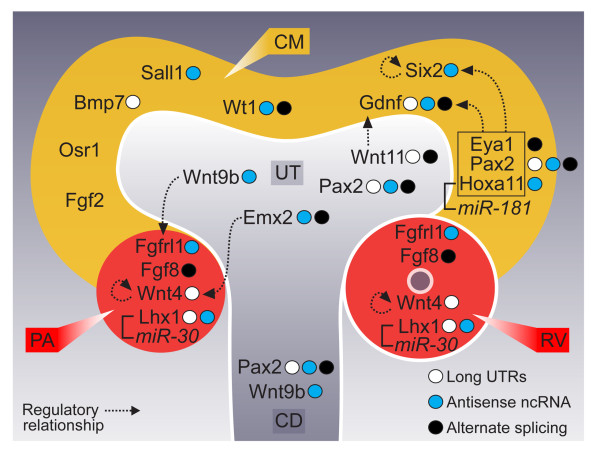
**Transcriptional complexity of the mesenchymal-epithelial transition network**. Transcriptional complexity associated with the 17 most characterized mesenchymal-epithelial transition pathway (MET) genes. Genes that have evidence of alternative splicing include alternate exon usage, alternate 5' and 3' exons highlighted with black circle. Genes with long 5' and/or 3' UTR signal are represented by white circles and antisense transcript in blue circles. Literature evidence of microRNA association is represented for *Lhx1 *(miR-30) and *Hoxa11 *(miR-181) along with other known transcriptional regulatory relationship (dotted arrows). Figure modified from Little et al [2].

Representations of biological networks and pathways typically report a gene as a single node, neglecting features of transcriptional complexity. To assess the extent of transcriptional complexity within kidney development networks, we surveyed the transcriptional complexity during MET program. This critical renal development event is paramount for normal renal function and disruption can alter nephron number which in turn predisposes individuals to kidney diseases [2]. A current review of kidney development describes 17 well-characterized loci [2] as being involved in this MET event. However, like many such reviews, this is gene-centric in nature. Our data shows extensive transcriptional complexity associated with all but two of the described MET developmental genes (Figure 6), and we have described the transcriptional landscape of this crucial biological process.

For eight loci with evidence for alternative exon usage, we scanned for changes in the protein domain composition to infer functional changes. Out of the four RefSeq canonical isoforms for *Fgf8*, two isoforms (variant 2 and 3) were detected in the kidney, which differed in presence or absence of exon 4 [67]. Removal of this exon excludes the signal-peptide normally associated with this growth factor, presumably leading to an intracellular protein with a different biological role. This may have implications for the formation of the renal vesicle, the first stage of nephron induction, where *Fgf8 *is expressed and has assumed to act as a secreted protein.

Alternative 5' ends were identified for the *Gdnf, Pax2, Eya1 *and *Wnt11 *loci. In humans, *EYA1 *is associated with three isoforms differing at the first exons [68]. In addition, RNA-Seq provided evidence for an additional uncharacterized exon between exon 1 and 2 of the canonical *Eya1 *RefSeq transcript EST tag evidence and gene models (Aceview: Eya1.fSep07). In the *Pax2 *locus, signal extending the 5' end as far as 10 kb provided compelling evidence for an alternative promoter signal beyond the current gene models.

Signal flanking 3' ends for genes such as *Pax2, Bmp7, Wnt4 *and *Lhx1 *mouse RefSeq models were supported by more complete gene models such as the human RefSeq transcripts and other gene prediction models. SISH validation of the observed *Lhx1 *and *Wnt4 *3' extensions confirms these events as an extension of the primary transcript and highlights the need for updated gene models.

Surprisingly, natural antisense transcripts were detected for 10/17 MET genes. Several antisense transcripts have previously been identified, such as *Emx2os *[66] and *Wt1AS *[62] where both antisense has been shown to positively regulate the respective sense transcript expression. SISH analyses of novel antisense expression for *Six2, Sall1 *and *Lhx1 *show concordant expression patterns with sense counterpart. Sense-antisense pairs identified for MET genes were arrayed in a head-to-head overlap at the 5' end which may be indicative of a bidirectional promoter, similar to *Wt1*-AS.

To infer candidate miRNAs involved in MET, we scanned the literature for MET genes with experimental evidence of miRNA target regulation. Only *Lhx1 *has been characterized as target of miR-30 within the context of kidney development [28]. Other MET genes have had characterized miRNA regulation in other tissue types, including regulation of *Hoxa11 *by miR-181 during muscle differentiation [69], and hypoxia-induced targeting of *Fgfrl1 *by miR-210 [70]. Such transcript-centric models reveal the undocumented layer of complexity associated with current models of regulatory networks which should be incorporated into functional validations studies.

## Discussion

Embryonic kidney development requires a high level of transcriptional co-ordination to form at least 25 known distinct cell types required to carry out specific renal functions. We have described here the first RNA-Seq profiling of whole embryonic mouse kidney and have integrated this information with previous microarray and SISH based atlases of expression during kidney development. What we show is that RNA-Seq offered detailed transcriptional profiling beyond the locus expression activity offered by most microarrays.

A major concern of whole organ profiling relates to the disproportional representation of all cell types in such complex cellular systems. Transcriptional profiling of whole organs using microarray has been problematic due to the heterogeneous tissue composition and proportions, which can overshadow differential gene expression of less abundant cell types [22]. Given the potentially unlimited dynamic range, RNA-Seq should overcome this hurdle. We demonstrate here that at sufficient depth, whole kidney transcriptome profiling by RNA-Seq can provide the resolution and coverage to detect over 99.7% of subcompartment-specific transcripts. Transcriptional output from each major subcompartment was also shown to be evenly distributed across the data based on subcompartment-specific transcript expression, with RNA-Seq detecting both abundant (above 10 RPKM) and low-level tissue-specific transcripts (below 1 RPKM). Despite this, it is important to note that the lack of normalization approaches for RNA-Seq, makes identification of rare, cell-type specific transcripts challenging, as highly expressed transcripts would obtain the most tag coverage.

The sensitivity of RNA-Seq makes whole organ profiling ideal for integration with pre-existing microarrays of kidney cell-types to achieve single nucleotide- and spatial- resolution of transcriptional complexity. Not all events detected in the RNA-Seq could be represented by Affymetrix probesets (i.e. alternative exon and 5' promoters) due to the 3'end bias of the Affymetrix 430.2 probeset design. The 3' end bias was instead ideal for survey of differential subcompartment localization of extended 3'UTRs and detecting occasional ncRNA transcript expression.

Overall, RNA-Seq profiling captured a wide range of transcriptional complexity during kidney development. These events were highlighted among a subset of well established kidney developmental genes throughout the study revealing new insights. For example, while alternative splicing of the *Wt1 *locus in the kidney has been extensively documented, we detected a uncharacterized mouse in-frame isoform without exons 4 and 5. This isoform was supported by the Ensembl mouse predicted transcripts but has only been reported in fish and turtles [34-36]. These two exons together encode a putative leucine zipper motif, located at the N-terminal region of *Wt1 *[34], which has been previously shown to contain protein-protein association domains [71]. This region allows *Wt1 *isoforms to self-associate, whereby removal of exon 4 and 5 would alter the dimerisation of WT1 protein isoforms and their ability to interact with other proteins [71].

The strand-specific nature of our RNA-Seq enabled sense-antisense transcript annotations. Although various techniques confirmed widespread presence in the mammalian genome [60,72,73], detection and identification of low abundance antisense transcripts, a common trait of antisense RNA, remained challenging due to sequencing depth limitations from these technologies [74]. The sequencing depth and strand-specific nature of RNA-Seq facilitated the use of a liberal approach for the identification of many sense-antisense transcripts including low-copy number antisense transcripts. In the analysis, several transcription factors critical for MET were associated with overlapping antisense ncRNA transcript expression. Many of these antisense ncRNA show syn-expression patterns with the sense pair as during SISH validation including the uncharacterized antisense for *Six2*, a marker of the renal progenitor cell population. The orientation is reminiscent of the *Wt1 *antisense (WT1AS), which has been shown to positively regulate *WT1 *protein expression levels [62] through a bidirectional promoter. Hence, this may also be true for the *Six2 *and *Sall1 *sense/antisense transcripts. Further functional validations will be required to determine antisense-mediated regulation for these key protein-coding genes.

MiRNAs have been shown to play an active role during embryonic development however individual miRNAs required for kidney development remains largely unexplored. To address this, the miRNA population from the embryonic kidney sample was isolated and sequenced to serve as a reference for the entire, embryonic kidney miRNA repertoire. Next, we associated subcompartment localization of miRNAs from intergenic pri-miRNA expression. We focused on Affymetrix probesets that directly overlapped with the embedded miRNA, which lead to the identification of miR-214 from the *Dnm3os *transcript. Both SISH riboprobe and Affymetrix probeset expression profiles detected expression in all kidney mesenchymal/interstitial subcompartments except cap mesenchyme, where it was detected during SISH but down-regulated in the microarray profile of the *Six2*+ cap mesenchyme population.

*Six2 *is a marker of the nephron progenitor population, which maintains progenitor renewal by preventing epithelial differentiation during MET. The inhibitory nature of miRNA, through miR-214, may reflect a role in suppressing self-renewal and therefore promoting differentiation. This hypothesis aligns with the previously described role of miR-214 as a promoter of cellular differentiation of skeletal muscle cells. miR-214 has also been shown to promote ES cell differentiation via the regulation polycomb group proteins [75] and by modulating Hedgehog signalling [76]. In the kidney, *Shh*, part of the Hedgehog signalling pathway, is required for mesenchymal proliferation and differentiation of smooth muscle progenitor cells [77]. This gene may also be regulated by miR-214.

Almost all the genes involved in the MET pathway show some form transcriptional complexity, which is largely unaccounted for during functional characterization of many of these loci. Hence, our findings now provide an opportunity to move towards transcript-centric models of biological pathways and networks in kidney organogenesis.

## Conclusions

In conclusion, this dataset provides a valuable resource with which to interrogate transcriptional control of kidney development. Integration of the RNA-Seq data with pre-existing resources such as tissue-specific microarrays and SISH provides a dynamic atlas of the spatial and transcriptional regulation of a developing organ, thereby representing an ideal baseline for comparative studies into kidney development abnormalities. Specifically, our analyses highlight new transcriptional components active during key stages of kidney development that can now be prioritized for further functional characterization.

## Methods

### Library Prep and Sequencing of mRNA and miRNA

Total RNA (10 ug) from 46 embryonic kidney (15.5 dpc) from 5 litters of CD1 mice was put through one round of poly (A) selection (Oligotex Kit, Qiagen) followed by ribosomal depletion (Ribominus Kit, Invitrogen) to select mRNA. The enriched mRNA was fragmented by digestion with RNaseIII (Ambion), and purified on a Microcon YM30 column (Microcon). Fragmented mRNA was used to generate libraries as specified in the Whole Transcriptome Analysis Kit (Ambion) protocol for mRNA and Short RNA Expression (SREK). The SREK library was barcoded (barcode: Series A, Applied Biosystems) and pooled. Emulsions PCR (8×) and large scale enrichment (LaSE) was carried out as outlined in the SOLiD 3 Plus template bead preparation manual. Sequencing was carried out on SOLiD system 3.5 and v3.5 chemistries to produce DNA sequence reads of 35-50(nt). Datasets available via the NCBI Short Read Archive (SRA026710).

### Mapping and Analysis

#### mRNA-Seq mapping

Mapping of SOLiD sequencing reads was performed using a recursive mapping strategy using RNA-MATE v1.1 [30] under default settings. Reads were mapped to the mouse genome (mm9) and a library of exon-exon junctions derived from gene models such as RefSeq, UCSC known genes, Ensembl, Aceview as previously detailed in [11]. Resulting mapped tags were presented as 'wiggle plots' (bedGraph data format) of tag abundance for visualization in UCSC Genome Browser. The mapped tag starts sites files from (the RNA-MATE output) were used to calculate tag frequency counts against RefSeq gene models.

#### RPKM normalization

Non-redundant RefSeq protein coding loci genomic co-ordinates was provided as BED files from the UCSC Genome Browser curation team. Tag start files were used to calculate expression as detailed in RNA-MATE manual. RefSeq gene reads per kilobases per million (RPKM) calculation was performed in Galaxy [24] and as detailed in [10].

#### Genome-wide identification of alternative exon and alternative 5' exon usage

A minimum of 2 tags were used to consider candidate alternate exon-exon junctions events overlapping RefSeq gene canonical junctions. As this produced a large list, we reduced the list to report only alternate exon-exon junction tags with ≥ 5 tags in Additional file 4. For alternative 5' exon usage, we used a stringent cutoff, ≥ 5 tags. This is to circumvent weaker signals in the 5' end arising from 3' bias arising from RNA-Seq protocols [7].

#### Extended 3' UTR

Tags mapping downstream of the 3'UTR boundary of RefSeq and UCSC Genes were analysed in 30 bp windows along a 20 kb (non-overlapping) radius. Presence of extended 3'UTR was calculated for genes above 1RPKM. Expression beyond the 3'UTR of RefSeq gene models were required to: a) be greater than 50% of the RPKM value b) have expression in any 10 consecutive 30 bp sliding window, and c) have expression extended greater than 500 bp.

#### Sense-Antisense transcripts

Antisense expression were annotated against RefSeq transcripts coordinated obtained from the UCSC Genome Browser (mm9). Antisense partners were required to have expression greater than 10 RPKM. Reads were required to map on the opposite strand of the RefSeq transcript, within the annotated coding or untranslated regions.

#### MiRNA-Seq mapping

Small RNA sequencing tags were aligned against miRBase v15 pre-miRNA hairpins using miRNA-MATE, an open source alignment tool designed in our laboratory specifically for colour-space miRNA analysis (http://grimmond.imb.uq.edu.au/miRNA-MATE/; manuscript in preparation). miRNA-MATE uses the recursive style of matching, described in Cloonan et al [30], for sensitive miRNA expression detection, but also can identify and strip the adaptor to determine the precise ends of the captured miRNAs. During alignment, up to 2 mismatches were allowed, treating valid-adjacent mismatches (those colour-space mismatches when located side-by side, indicate the presence of a single nucleotide variant) as a single mismatch.

#### Comparisons against Affymetrix probesets

Probesets were created from a consensus sequence obtained from NetAffx [78]. The consensus sequence was mapped to the mm9 genome using blat using default parameters. Scoring of an alignment is based on UCSC Genome Browser Guidelines [79]. If a consensus sequence matches two or more locations with the same highest score, both multi-mapping consensus sequences were included. Individual probes from each Affymetrix probeset was mapped to a library of consensus probeset sequence obtained from NetAffx. Probesets were then represented onto the genome based on the consensus sequence mapping coordinates results.

### Riboprobe design and generation

The complete protocol for digoxigenin (Dig)-labeled riboprobe synthesis is available and described in detail on the GUDMAP gene expression database [80]. Primers were ordered from Invitrogen and were designed to amplify a 3' UTR region of the RIKEN Fantom3 cDNA clone models, between 500 and 800 bp. Riboprobes were amplified from 15.5-dpc whole embryonic mouse cDNA. The 3' primers were tagged with a T7 polymerase (Roche), for in vitro transcription of Dig-labeled riboprobes. Riboprobes were then purified with lithium chloride precipitation and stored at -20°C overnight. Samples were then spun for 20 min at 4°C with supernatant discarded after the spin, gently washed with of chilled 70% ethanol, and then spun at 4°C. Supernatants were discarded and samples dried for 10 min at room temperature where pellets were then resuspended with 25 μl of water and stored at -70°C.

### Section *in situ *hybridization validations

The complete protocol for section in-situ hybridization (SISH) is available and described in detail on the GUDMAP gene expression database [80]. For *Dnm3os*, manual SISH was performed using NTM-based dye. The complete protocol is described in [81]. Briefly 7 um paraffin sections of 15.5 dpc CD1 mouse kidneys incubated in 10 ug/ml proteinase K for 20 mins at room temperature. Next, samples were washed and refixed with 4% paraformaldehyde for 10 mins at room temperature. This is followed by acetylation and pre-hybridization using hybridization solution for 2 hrs at room temperature. Hybridization was carried out overnight at 60°C. Slides were then washed by NT buffer at room temperature before incubating for 2 h with blocking solution in a humidified chamber. A 1:1000 dilution of anti-digoxigenin antibody (Roche Applied Science) in blocking solution was added to the slides and incubated overnight at 4°C. Unbound antibodies were removed by washing in NT buffer. Sections were equilibrated in NTM buffer and incubated in color solution until purple staining was satisfactory.

### Quantitative RT PCR

To validate *Wt1 *splice event (spliced exons 4 and 5) detected from the RNA-Seq data, the mRNA levels of the uncharacterized event was compared against a well characterized splice event (spliced exon 5) of *Wt1*. PCR was performed in quadruplicates using matched sample that was used to generate the RNA-Seq cDNA libraries. Samples were run with Actin housekeeping gene as a positive control. Primers were designed to span across exon junctions 3 and 6 junctions (Kidney_Wt1_minus exons 4 and 5) (Forward: CCCCTACTGACAGTTGCACA; Reverse: TACTGGGCACCACAGAGGAT). As a control, primers were also designed for a known *Wt1 *splice event (Kidney_Wt1_ctrl minus exon 5 (known)) (Forward: CTTGAATGCATGACCTGGAA; Reverse: TACTGGGCACCACAGAGGAT). Relative mRNA expression of Kidney_Wt1_minus exons 4 and 5 was compared to the "known" event and reported as relative mRNA fold abundance.

## Ethics statement

All animal work contributing to this manuscript was conducted according to all state, national and international guidelines. Animal ethics approval was provided by AEEC3 of The University of Queensland (Approval IMB/572/08/NIH (NF)).

## Authors' contributions

RDT and SMG conceived the idea. RDT analyzed the data, designed the primers, riboprobes and performed the ISH validations, and wrote the manuscript. NC mapped the data and edited the manuscript. BBG and SW prepared the cDNA libraries for sequencing. BBG, EN, and SW sequenced the samples. TRM performed the sense-antisense mappings. GK provided scripts to perform alternative exon, alternative 5' exon, and extended 3'UTR analysis. KK performed the qRT-PCR validations. KMG annotated the ISH images and provided Figure 6. BAR provided 15.5 dpc CD1 mice kidney samples and assisted with the ISH validations. DT compiled the UCSC custom Affymetrix heatmap tracks and performed Affymetrix probe mappings. HSC assisted with ISH. JAS formatted the datasets for submission. JSM edited the manuscript. MHL provided insights for the alternative splicing analysis, the interpretation of the gene expression within the developing kidney and edited the manuscript. SMG organized the project and edited the manuscript. All authors read and approved the final manuscript.

## Supplementary Material

Additional file 1 **RPKM for RefSeq loci**. RPKM calculation and tag abundance of non-redundant RefSeq loci (RefSeq loci compiled by UCSC Genome Browser). Click here for file

Additional file 2 **Overlapping antisense expression for *Lhx1***. UCSC screenshot of *Lhx1 *(negative strand) and antisense expression (positive strand). Previously unassigned Affymetrix probe 1439232_at aligned with overlapping (head-to-head) antisense transcript *1500016L03Rik *with corresponding heatmap of kidney subcompartment expression. Microarray compartments from top to bottom of heatmap: ureteric tip; s-shaped body; proximal tubule; cortical, and medullary interstitium; medullary, and cortical collecting duct; renal corpuscle; cap mesenchyme; loop of Henle; renal vesicle. Click here for file

Additional file 3 **Extended 3' UTR signal**. Transcripts with tags beyond annotated 3'UTR within a 20 kb window. Click here for file

Additional file 4 **Alternative Exon Junctions (> 5 tags)**. Loci with alternative splicing supported by a minimum of 5 exon junction tags. Click here for file

Additional file 5 ***Ret *isoforms**. UCSC screen shot of *Ret *locus. RefSeq gene model representation of *Ret *isoforms *Ret51 *(top) and *Ret51 *(bottom). Difference within the C-terminal end of gene is captured by RNA-Seq exon junction tags and signal. Microarray compartments from top to bottom of heatmap: ureteric tip; s-shaped body; proximal tubule; cortical, and medullary interstitium; medullary, and cortical collecting duct; renal corpuscle; cap mesenchyme; loop of Henle; renal vesicle. Click here for file

Additional file 6 **mRNA expression level measured by qRT-PCR for Wt1 splice events**. Kidney_Wt1_ctrl minus exon 5 (known) represents a previously well characterized Wt1 splice event where exon 5 has been spliced out. Kidney_Wt1_minus exons 4 and 5 (Ensembl transcript: ENSMUST00000111100) represents uncharacterized splice event where exons 4 and 5 are spliced out. The expression ratios were averaged from quadruplicates runs. Kidney_Wt1_minus exons 4 and 5 was compared against the "known" splice event which shows that the minus exons 4-5 event is expressed at a higher level than the "known" event. Click here for file

Additional file 7 **Alternative Exon Junctions (> 5 tags)**. Exon junction tags with reference (UCSC Genes) or non-reference (other models) evidence of alternative 5' end usage. Click here for file

Additional file 8 **Alt. 5 prime junction tags**. Loci with alternative splicing supported by a minimum of 5 exon junction tags. Click here for file

Additional file 9 **Embryonic kidney microRNAs**. Tag abundance of mature miRNAs based on mapping to hairpins (mirBase version 15). Click here for file

Additional file 10 **Kidney Primary miRNA (pri-miRNA) transcripts**. Intergenic pri-miRNA annotations from [51]*with *corresponding miRNAs expressed in 15.5 dpc kidney. Affymetrix probeset ID's representing pri-miRNA are also provided. Click here for file

Additional file 11 **Sense and Antisense transcripts**. Antisense transcripts overlapping RefSeq transcripts detected in developing mouse kidneys. Click here for file
