# Supplementary figures and images for: Refining transcriptional programs in kidney development by integration of deep RNA-sequencing and array-based spatial profiling
Source: BMC Genomics. 2011 Sep 5;12:441. doi: 10.1186/1471-2164-12-441 (PMC3180702; doi:10.1186/1471-2164-12-441)

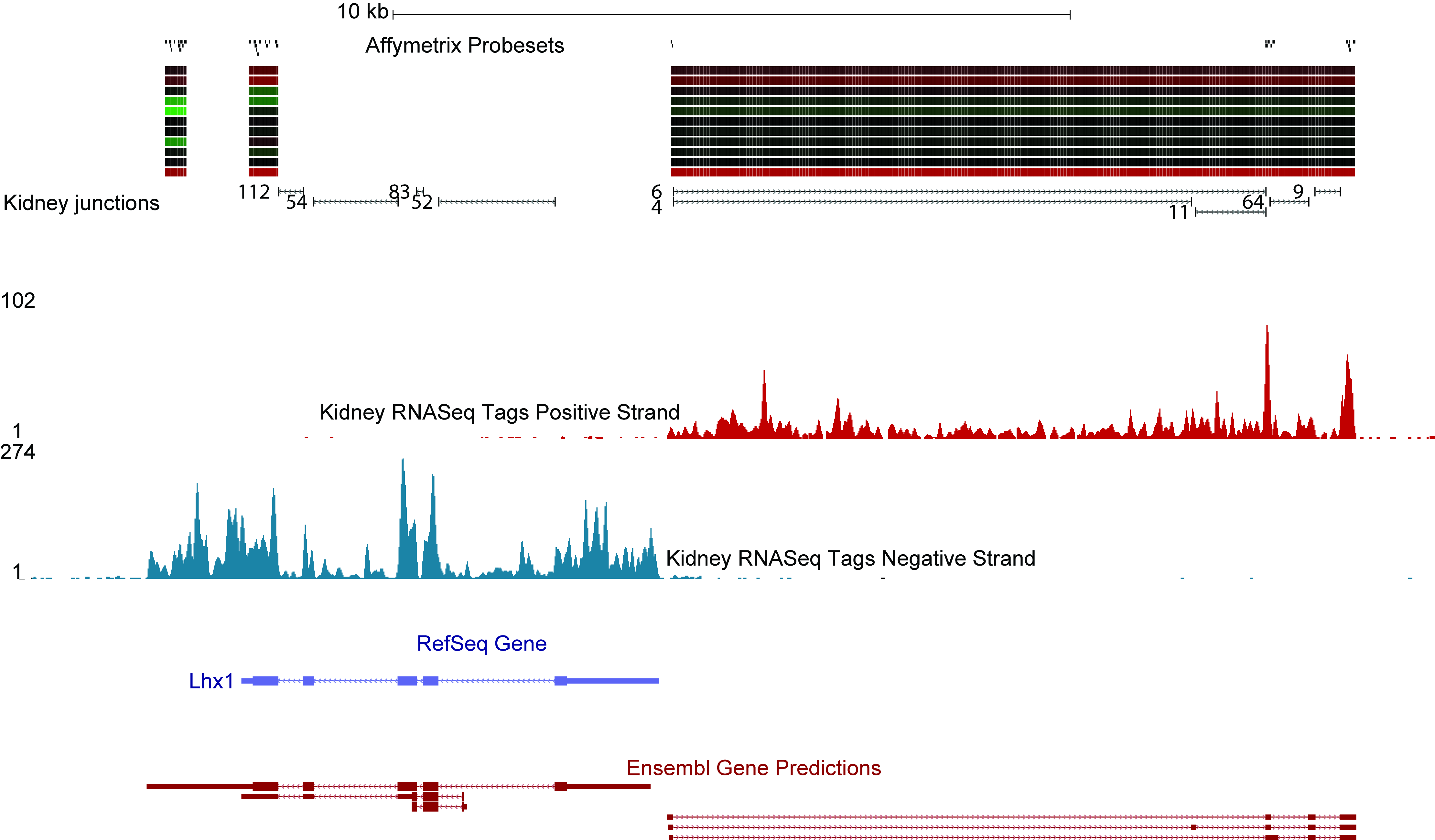

Supplement: Additional file 2 — Overlapping antisense expression for Lhx1. UCSC screenshot of Lhx1 (negative strand) and antisense expression (positive strand). Previously unassigned Affymetrix probe 1439232_at aligned with overlapping (head-to-head) antisense transcript 1500016L03Rik with corresponding heatmap of kidney subcompartment expression. Microarray compartments from top to bottom of heatmap: ureteric tip; s-shaped body; proximal tubule; cortical, and medullary interstitium; medullary, and cortical collecting duct; renal corpuscle; cap mesenchyme; loop of Henle; renal vesicle. [file 1471-2164-12-441-S2.TIFF]

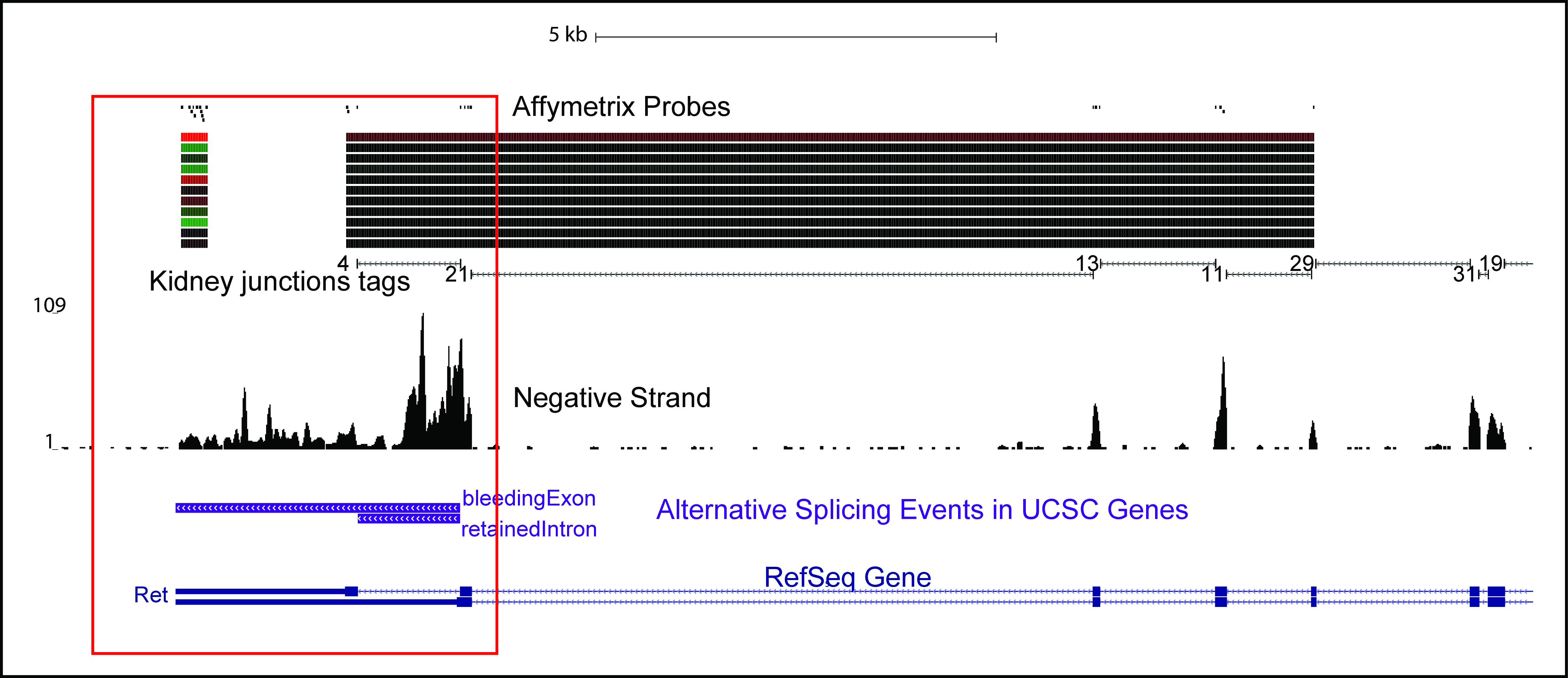

Supplement: Additional file 5 — Ret isoforms. UCSC screen shot of Ret locus. RefSeq gene model representation of Ret isoforms Ret51 (top) and Ret51 (bottom). Difference within the C-terminal end of gene is captured by RNA-Seq exon junction tags and signal. Microarray compartments from top to bottom of heatmap: ureteric tip; s-shaped body; proximal tubule; cortical, and medullary interstitium; medullary, and cortical collecting duct; renal corpuscle; cap mesenchyme; loop of Henle; renal vesicle. [file 1471-2164-12-441-S5.TIFF]

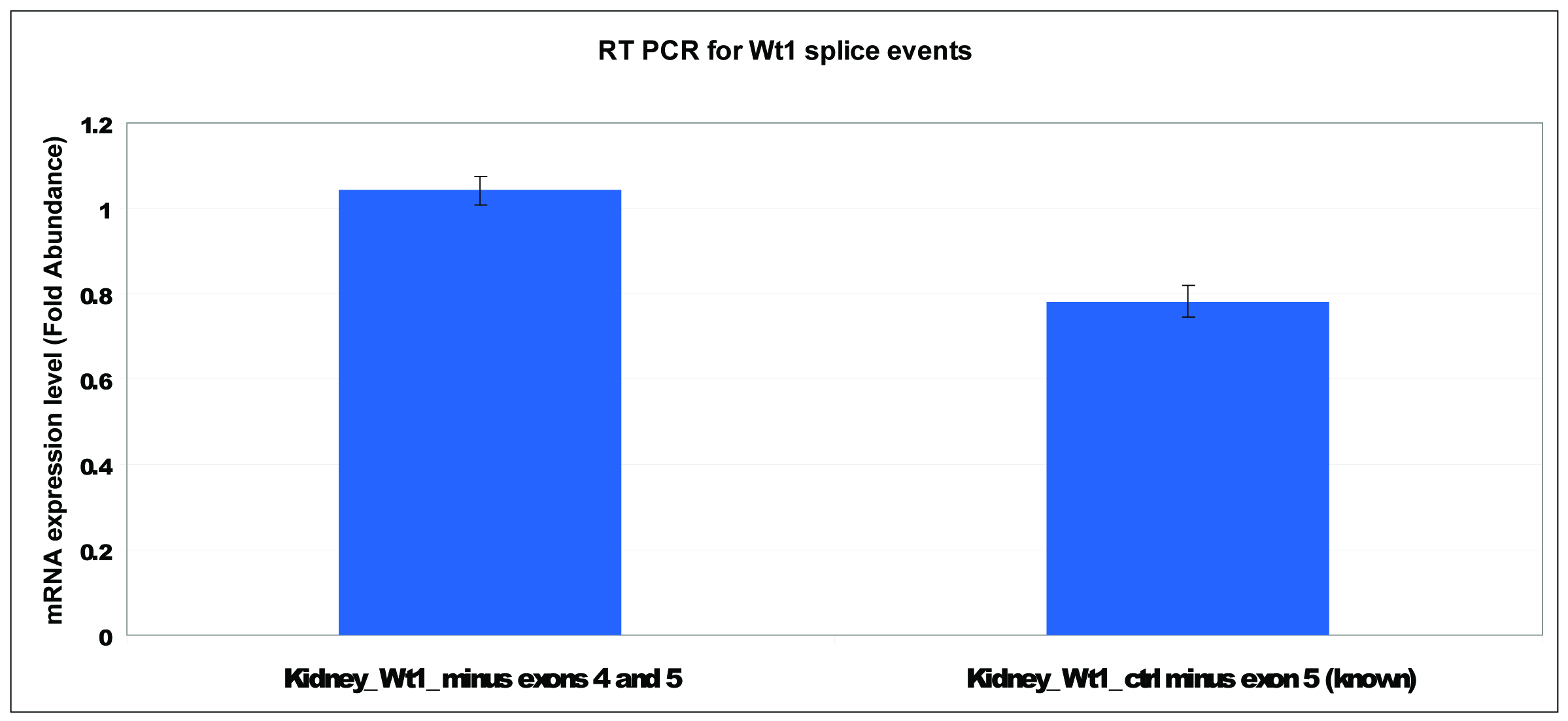

Supplement: Additional file 6 — mRNA expression level measured by qRT-PCR for Wt1 splice events. Kidney_Wt1_ctrl minus exon 5 (known) represents a previously well characterized Wt1 splice event where exon 5 has been spliced out. Kidney_Wt1_minus exons 4 and 5 (Ensembl transcript: ENSMUST00000111100) represents uncharacterized splice event where exons 4 and 5 are spliced out. The expression ratios were averaged from quadruplicates runs. Kidney_Wt1_minus exons 4 and 5 was compared against the "known" splice event which shows that the minus exons 4-5 event is expressed at a higher level than the "known" event. [file 1471-2164-12-441-S6.TIFF]
